# Improving spectacle wear in school children

**Published:** 2017-08-07

**Authors:** Priya Morjaria, P Dinesh Raj, GVS Murthy

**Affiliations:** 1 Research Fellow/Public Health Optometrist International Centre for Eye Health, London, UK.; 2 Project Coordinator: Indian Institute of Public Health, Hyderabad India.; 3 Director: Indian Institute of Public Health, Hyderabad, India & Professor Public Health Eye Care & Disability, LSHTM, London, UK.

**Spectacle compliance is low in many school eye health programmes. There are various reasons for this, including that children do not perceive a beneficial improvement in their vision. Accurate visual acuity (VA) measurement, refraction and prescribing based on the degree of improvement in VA can also help.**

**Figure:**
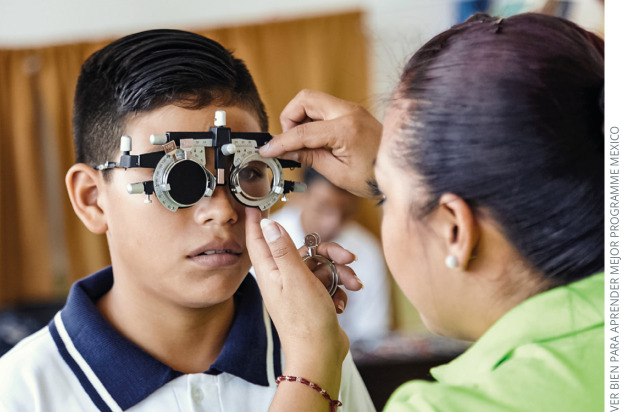
Inserting corrective trial lenses into a trial frame suitable for children. MEXICO

## Measurement of visual acuity

Children who fail visual acuity screening must undergo thorough and detailed visual acuity measurement. This is the first step in identifying those who may benefit from spectacle correction.

Ideally, visual acuity measurement is done in the school immediately or very soon after vision screening. This should be done by an optometrist or a trained refractionist experienced in measuring visual acuity in children. Visual acuity measurements carried out in schools should be of the same standard as at an eye unit.

The distance between the screener and the student (usually 6 metres) should be measured and marked appropriately. The student's chair may move around, so make a mark on the floor where the front legs of the chair should be. Check that the chair is in the right place before assessing each child. If using a standard, unlit visual acuity chart, the room should be well lit, taking care to avoid reflections off the chart. Backlit charts can be used in a darkened room. Students should not be distracted by strong external sources of light. Before starting to measure visual acuity, the optometrist/refractionist should check that the environment is correctly set up by sitting where the student will sit.

The following equipment is required:

- Tape measure
- Full tumbling E chart (or multi-letter Snellen). Ideally, this should be the logMAR version
- Eye occluder or a piece of card to place over one eye
- Student record sheet.

## Procedure

- Explain the test to the child. If an E chart is being used, ensure that they understand what they are being asked to do before starting to measure their visual acuity.
- Measure the acuity one eye at a time, usually the right eye first, then the left.
- If a child already wears spectacles, measure their acuity without spectacles first.
- Ensure that the chart is at the student's eye level.
- Cover the left eye with the eye occluder or a piece of card. It is advisable that they do not use their hand as they may be able to see between their fingers.
- If using a tumbling E chart, point first to the 6/60 size E and ask the student to indicate which way the bars of the E point. Proceed down the chart, pointing out each E in turn, taking care not to cover any part of the E with the pointer.
- Follow the same procedure if using a standard letter Snellen chart.
- To see any particular line of the chart, the child must be able to see at least three of the five Es or letters.
- The smallest line accurately read is expressed as a fraction, e.g. 6/18. The upper number refers to the distance between the chart and the person being tested (6 metres), and the lower number is the line on the E or Snellen chart that the child can see.
- Record the VA for each eye immediately after measuring the acuity, stating whether this was tested with or without spectacle correction.
- If the child cannot read the 6/60 E or letter this is recorded as <6/60.

## Refraction

Refraction should be undertaken by a competent practitioner experienced in refracting children.

**NOTE: Children whose visual acuity does not improve to normal with refraction must be referred for examination to determine the cause so that appropriate action can be taken.**

Retinoscopy, or preliminary assessment using an autorefractor appropriate for children, should be followed by comprehensive subjective refraction. Children should be referred for cycloplegic refraction if they are uncooperative, if there is a variable or inconsistent end-point to refraction, in the presence of strabismus or suspected amblyopia and if they are difficult to refract because of media opacities or irregular corneas.

Before describing how to prescribe spectacles for children, it is important to understand why children may not wear their spectacles.

## Why children do not wear their spectacles

A key issue in school eye health programmes is that children do not always wear the spectacles provided, which means they do not benefit when they have the potential to. Studies in all income settings show that spectacle wear is often less than 50%. Reasons include:

1. Parents do not buy the spectacles
2. Parents are concerned about their child's appearance
3. Parents are concerned that spectacles will weaken their child's eyes
4. Teachers do not encourage children to wear their spectacles
5. The child is teased or bullied for wearing spectacles
6. The child does not like the spectacles or they are uncomfortable
7. The child does not notice any improvement in vision

There are simple solutions for many of these reasons.

- Reasons 1–5 can be addressed by health education which should include teachers, parents and all children whether they need spectacles or not.
- Reason 6 can be addressed by ensuring that children select the frames they prefer from a range of colours and designs which school children in the programme area say they like, and by checking that the frames are a good fit.
- Reason 7 relates to prescribing.

A recent randomised clinical trial compared rates of spectacle wear in children based on the type of spectacles used. In the trial, children received spectacles only if doing so improved their visual acuity by two or more lines. When followed up after 3–4 months, 75% of all the children were still wearing their spectacles or had them at school.^1^

This is much higher than in other studies, conducted among children of similar ages, in which prescribing was based on the degree of refractive error found at retinoscopy. This meant that spectacles were prescribed even when some children already had good VA in one eye. These children would not notice an improvement in their vision and would be less likely to wear their spectacles.

## Prescribing guidance

The prescribing guidelines given here are based on those followed in the clinical trial^1^, in modified form. We hope that the guidelines will help to avoid unnecessary prescribing of spectacles – which will not be worn – in settings with limited resources. However, this must not override the needs of an individual child. **Note:** The guidelines apply to children with VA <6/9.

Correction for myopia is indicated if:

- Minus powered lenses improve the VA by 2 or more logMAR (or Snellen) VA lines in the better eye or with both eyes tested together.

Correction for hypermetropia is indicated if:

- Plus powered lenses improve the acuity by 2 or more logMAR (or Snellen) VA lines in the better eye or with both eyes tested together, and/or noticeably improve eye comfort when reading
- There is amblyopia (and the child's age suggests that the amblyopia is potentially treatable)
- There is esotropia or a large esophoria (and the child has some potential for normal binocular vision).

**Figure:**
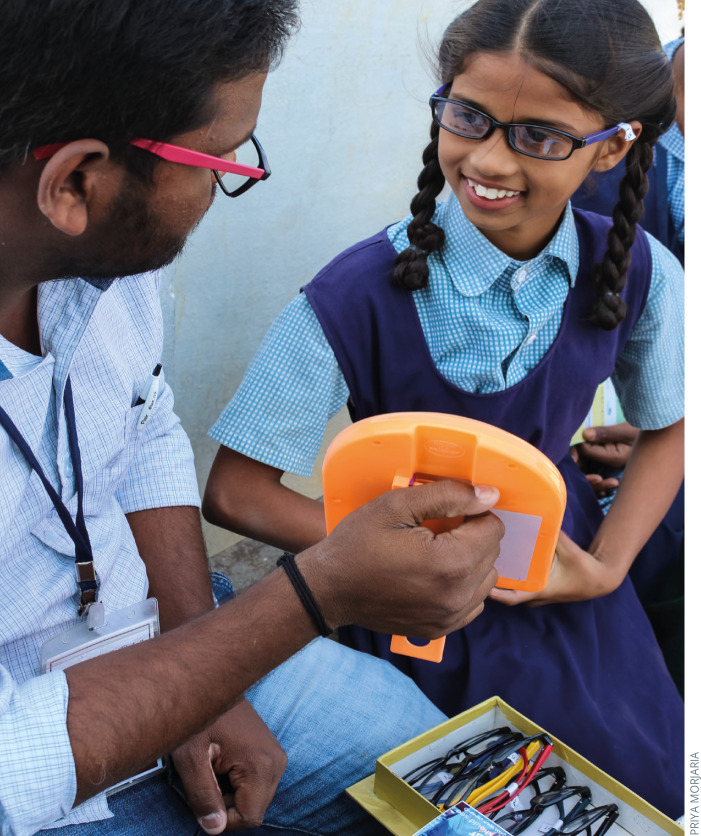
Children are more likely to wear their spectacles if they are happy with their appearance. INDIA

Correction of astigmatism is indicated if:

- Cylindrical lenses improve the acuity by 2 or more logMAR (or Snellen) VA lines in the better eye or with both eyes tested together; and/or noticeably improve eye comfort
- There is amblyopia (and the child's age suggests that the amblyopia is potentially treatable).

Correction for anisometropia is indicated if:

- There is significant anisometropia (i.e. 1D or more), **and** one or more of the following apply:
  – correctly balanced lenses improve the acuity of the most affected eye by 2 or more logMAR VA lines
  – eye comfort is notably improved.
- There is amblyopia (and the child's age suggests that the amblyopia is potentially treatable).

## Conclusion

There is increasing evidence that, if most children see better with spectacles than without, a higher proportion will wear them. Ideally, a sample of children who do not their wear spectacles should be interviewed to find out why they are not wearing them so that corrective measures can be put in place. An important measure of success for any school eye health programme is the proportion of children given spectacles who subsequently wear them - it is not enough just to measure and report the number of spectacles that are dispensed.
